# Supplementary material for: How human factors affect escalation of care: a protocol for a qualitative evidence synthesis of studies
Source: BMJ Open. 2019 Apr 8;9(4):e025969. doi: 10.1136/bmjopen-2018-025969 (PMC6500321; doi:10.1136/bmjopen-2018-025969)
Supplement: Author's manuscript [file bmjopen-2018-025969.draft_revisions.pdf]

BMJ Open

BMJ Open is committed to open peer review. As part of this commitment we make the peer review history of every article we publish publicly available.

When an article is published we post the peer reviewers' comments and the authors' responses online. We also post the versions of the paper that were used during peer review. These are the versions that the peer review comments apply to.

The versions of the paper that follow are the versions that were submitted during the peer review process. They are not the versions of record or the final published versions. They should not be cited or distributed as the published version of this manuscript.

BMJ Open is an open access journal and the full, final, typeset and author-corrected version of record of the manuscript is available on our site with no access controls, subscription charges or pay-per-view fees (http://bmjopen.bmj.com).

If you have any questions on BMJ Open's open peer review process please email info.bmjopen@bmj.com

BMJ Open

How human factors affect escalation of care: a protocol for a systematic review and thematic synthesis of qualitative studies

Journal:	*BMJ Open*
Manuscript ID	bmjopen-2018-025969
Article Type:	Protocol
Date Submitted by the Author:	10-Aug-2018

Complete List of Authors:	Ede, Jody; John Radcliffe Hospital, University of Oxford<br>Westgate, Verity ; University of Oxford, Nuffield Department of Clinical Neurosciences<br>Darbyshire, Julie; University of Oxford, NDCN<br>Petrinic, Tatjana; University of Oxford Health Care Libraries, Cairns Library<br>Watkinson, Peter; Oxford University Hospitals NHS Trust, Kadoorie Centre for Critical Care research and Education

Keywords:	Failure to rescue, Escalation of care, Human factors, Qualitative, Thematic syntheisis, Systematic Review

SCHOLARONE™
Manuscripts

Title Page

How human factors affect escalation of care: a protocol for a systematic review and thematic synthesis of qualitative studies

Corresponding Author Jody Ede University of Oxford Jody.ede@ndcn.ox.ac.uk

Verity Westgate University of Oxford verity.westgate@ndcn.ox.ac.uk

Tatjana Petrinic Oxford University Hospital NHS Trust tatjana.petrinic@bodleian.ox.ac.uk

Julie Darbyshire University of Oxford Julie.darbyshire@ndcn.ox.ac.uk

Peter Watkinson Associate Professor University of Oxford peter.watkinson@ndcn.ox.ac.uk

“Supported by the NIHR Biomedical Research Centre, based at Oxford University Hospitals Trust, Oxford and the Wellcome Trust through the Health Innovation Challenge Fund. The views expressed are those of the author(s) and not necessarily those of the NHS, the NIHR or the Department of Health”

Word count: 1264

Keywords: Failure to rescue, escalation of care, human factors, qualitative, thematic, systematic

For peer review only

For peer review only - http://bmjopen.bmj.com/site/about/guidelines.xhtml

ABSTRACT

Introduction

Failure to rescue (FTR) is defined as mortality after complications during an in-hospital admission. Incidence of FTR varies between hospitals but has been estimated as 10.9% in high-volume hospitals and 13.3% in low-volume hospitals. Several national reports such as National Confidential Enquiry into Patient Outcomes and Death (NCEPOD) and NICE CG 50 emphasise this theme

For FTR to be avoided, there must be a successful escalation of care (EOC) initiated by bedside staff. Studies have found that Human Factors such as situational awareness, team working, communication and safety culture contribute to FTR. Understanding these human factors is essential to developing working systems that mitigate barriers and encourage facilitation of EOC. This qualitative systematic review is the first synthesis of what is known about the human factors that affect EOC.

Methods and Analysis

We will search MEDLINE (Ovid) and EMBASE (Ovid) for studies describing human factors that affect FTR and EOC. A search strategy was developed by two researchers assisted by a medical librarian. Only studies exploring EOC in hospital ward populations using qualitative data collection methods will be included. Screening will be conducted by two researchers from different professional backgrounds. Selected studies will be assessed for quality, rigor and limitations. Two researchers will extract and thematically synthesise codes using a piloted data extraction tool to develop analytical themes.

Ethics and dissemination

This systematic review will use available published literature and therefore no ethical approval is required. This systematic review will be limited by the quality of studies available and the rigor and reproducibility of study findings. This review will synthesise what is known about human factors and escalation of care, highlighting gaps within the literature. Results of this review will be published in peer reviewed journal, presented at conferences and publicised on social media.

TRIAL REGISTRATION

PROSPERO: (CRD42018104745)

ARTICLE SUMMARY

Strengths and Limitations of this study

- FTR is a common and significant problem in healthcare which affects patient mortality
- For FTR to be avoided, an escalation of care needs to occur. This efficacy of this can be positively or negatively affected by human factors
- This protocol ensures a comprehensive and unbiased search and analysis of qualitative studies exploring this phenomenon using best practice guidelines
- The results of this review will identify strengths and weaknesses of the literature in this area
- This review will highlight future research direction and address some of the identified weaknesses

For peer review only - http://bmjopen.bmj.com/site/about/guidelines.xhtml

For peer review only

INTRODUCTION

FTR is defined as the mortality rate of patients who suffer complications in hospital (1) . The incidence of FTR varies between hospitals but has been estimated as 10.9% in high-volume hospitals and 13.3% in low-volume hospitals. A proportion of patient deaths (32%) reported to the National Patient Safety Agency (NSPA) had failures surrounding diagnostic errors and deteriorations which were not adequately recognised (5). This theme is present in several national reports such as National Confidential Enquiry into Patient Outcomes and Death (NCEPOD) (3,6–8) and NICE CG 50 (4).

For FTR to be avoided, bedside clinical staff must usually initiate successful escalation of care (EOC) (9). This staged process requires detection of deterioration, communication about deterioration, and medical actions following a senior review (3). Many factors affect this process such as situational awareness, team working, communication, safety culture and leadership (3,10–14). Understanding these human factors is essential to developing working systems that mitigate barriers and encourage facilitation.

As a primary outcome, this qualitative systematic review will identify the human factors which affect EOC in the acute hospital setting. It will summarise what is currently understood about the involvement of human factors and their implications for practice. As a secondary outcome, it will identify any gaps in the current literature and establish strengths and weaknesses of the research. This will identify potential areas for further research in human factors and EOC.

Methods and Analysis

Registration

This protocol adheres to the requirements of Preferred Reporting Items for Systematic Review and Meta-analysis Protocols (PRISMA-P). The protocol was registered with PROSPERO (CRD42018104745)

Information sources

Literature search strategies will be developed using Medical Subject Headings (MeSH) and text words related to the human factors involved in the escalation of care for deteriorating patients.

The following databases will be searched: MEDLINE (Ovid), EMBASE (Ovid).

Reference lists of eligible studies and relevant reviews will be explored to identify further eligible studies.

Search strategy

A draft of the search strategy was developed by three of the authors (JE, VW and TP - a medical librarian). The proposed search strategy is shown in the online Supplementary File 1.

Inclusion Criteria

Types of studies
This systematic review will include qualitative studies which report primary data. Qualitative studies are defined as those studies which use qualitative data collection and analysis methods. These can

For peer review only - http://bmjopen.bmj.com/site/about/guidelines.xhtml

include but are not limited to: ethnography, interviews, focus groups and human factors methods. Data analysis is likely to be but not limited to: thematic analysis, grounded theory and discourse analysis.

Phenomenon of interest

Studies must report primary data and describe the human factors which affect FTR and EOC. FTR is defined as patient mortality following complications (1) and EOC is a staged process where patients are detected as deteriorating and that deterioration is communicated followed by a senior review (3). We will include any qualitative study which explores the perspective of patients or clinical staff (adults or paediatric) and the human factors which affect the EOC process. We are defining human factors as any barrier or facilitator that affects teamwork, tasks, equipment, workspace, culture and organisation (15).

Setting

The study setting is in-hospital, ward care.

Exclusion criteria

Types of studies

We will exclude systematic reviews, grey-literature, editorials, letters, practice guidelines and abstract-only reports. We will also exclude protocols without study data.

Phenomenon of interest

We are only interested in real-life scenarios where human factors effects can be studied in the patient environment. Simulation based studies will be excluded.

Setting

We will exclude studies carried out in the Emergency Department, Critical Care (including the Intensive Care Unit and Coronary care) or Maternity. These are specialised areas which makes generalisability of EOC themes to the ward environment challenging. We will also exclude studies set in palliative care.

Time-frame

No time limitations will be applied

Language

Non-English papers will be excluded.

Study selection

Reference lists from both databases will be entered into Covidence software (Covidence systematic review software, Veritas Health Innovation, Melbourne, Australia. Available at www.covidence.org). Papers will be de-duplicated. Two authors will independently screen titles and abstracts of identified papers against the inclusion and exclusion criteria. They will not be blinded to journal titles, study authors or institutions. If there is disagreement or uncertainty regarding eligibility, the full-text will be reviewed. We will retrieve full-text for all articles not excluded by the initial screening. Two authors will independently assess these papers against the inclusion and exclusion criteria outlined above. We will resolve disagreements about eligibility by discussion between the screening researchers or a third party. We will record the reason for excluding studies.

Data extraction

For peer review only - http://bmjopen.bmj.com/site/about/guidelines.xhtml

For peer review only

Data extraction tools will be developed and piloted before the review takes place. Extracted data will be entered into an Excel spreadsheet. Initial codes from studies will be documented with NVivo [NVivo qualitative data analysis Software; QSR International Pty Ltd. Version 10, 2014]. Two reviewers will independently extract a selection data from the texts to ensure validity of results. Any discrepancies within the data collection phase will be resolved by discussion between reviewers.

Data items extracted

We will extract the following data from each included publication. The data extraction method has been piloted with a sample selection of papers and valid data has been obtained.

Table 1- Anticipated data to be extracted

Study Characteristics	Patient/Participant demographics	Study setting	Themes	Rigor
• Author<br>• Date of study<br>• Study Type<br>• Methodology<br>• Country of study<br>• Data collection methods<br>• Journal<br>• Data analyses	• Age<br>• Patient group<br>• In-patient characterisation	• Level of care<br>• Hospital Type<br>• Education	• Codes	• Strengths<br>• Weaknesses<br>• Reporting guidelines used

Quality Assessment

The CASP qualitative checklist (Critical Appraisal Skills Programme) will be used to assess credibility, transferability, dependability and confirmability. This checklist is an extensive and comprehensive tool commonly used in qualitative study assessment (16,17). Two researchers will discuss quality findings for each study and a consensus will be reached as to the studies' inclusion or exclusion within the systematic review. As part of the CASP assessment the authors will explore the potential for reporting bias within the studies and biases will be reported in studies' limitations.

Data Analysis

We will undertake a thematic synthesis (17) using the Thomas and Harden (18) framework. The three stages of the framework are: coding of the findings of studies, categorisation of codes into descriptive themes, and categorisation of descriptive themes into analytical themes (19). NVivo software will be used to facilitate the analysis and record decisions (audit trail) of coding by the researchers. Codes relating to human factors and EOC will be identified in the text, and tables will be used to create descriptive and analytical themes. Key codes, descriptive themes and analytical themes will be presented in the results.

Ethics and dissemination

The proposed systematic review will use available published literature and therefore no ethical approval is required. This systematic review will be limited by the quality of studies available and the rigor and reproducibility of study findings. Original studies included in the review could themselves be limited and it may be difficult to assess the researcher involvement and their individual bias. The

For peer review only

For peer review only - http://bmjopen.bmj.com/site/about/guidelines.xhtml

two researchers carrying out screening for this review come from different professional backgrounds; this limits interpretation bias when assessing the studies to include. A recognised assessment tool will be used to determine study quality. Using NVivo to code studies will aid transparency in and demonstrate a clear strategy for identifying themes. An audit trail will be kept throughout the systematic review detailing research decisions made and methodological steps taken.

The results from this review will be published and made publically available. A number of social media techniques (Twitter, Facebook) will be used to promote the protocol, final systematic review paper and results. We will also aim to attend at least one conference to present findings from this work.

For peer review only

For peer review only - http://bmjopen.bmj.com/site/about/guidelines.xhtml

References

1. Ghaferi AA, Osborne NH, Birkmeyer JD, Dimick JB. Hospital characteristics associated with failure to rescue from complications after pancreatectomy. J Am Coll Surg [Internet]. 2010;211(3):325–30. Available from: http://dx.doi.org/10.1016/j.jamcollsurg.2010.04.025

2. Gonzalez AA, Dimick JB, Birkmeyer JD, Ghaferi AA. Understanding the volume-outcome effect in cardiovascular surgery: The role of failure to rescue. JAMA Surg. 2014;149(2):119–23.

3. Findlay G, Shotton H, Mason K. Time to Intervene? Natl Confid Enq into Patient Oucome Death [Internet]. 2012;1–125. Available from: http://www.ncepod.org.uk/2012report1/downloads/CAP_fullreport.pdf

4. NICE. Acutely ill adults in hospital: recognising Acutely ill adults in hospital: recognising and responding to deterior and responding to deterioration (CG50). 2007;(July). Available from: www.nice.org.uk/guidance/cg50

5. NHS Improvement. The adult patient who is deteriorating: sharing learning from literature, incident reports and root cause analysis investigations. 2016;(July):23. Available from: https://improvement.nhs.uk/documents/176/Deterioration_in_adults_report_7july.pdf

6. Fox P. An acute problem. Nurs Elder. 2005;3(4):33.

7. Harper SJ. NCEPOD: Caring to the end? J Intensive Care Soc. 2010;11(2):88–9.

8. National Confidential Enquiry into Patient Outcome and Death. Emergency Admissions: A jouney in the right direction? 2007; Available from: http://www.ncepod.org.uk/2007ea.htm

9. Johnston M, Arora S, King D, Stroman L, Darzi A. Escalation of care and failure to rescue: A multicenter, multiprofessional qualitative study. Surgery [Internet]. 2014 Jun [cited 2017 Aug 7];155(6):989–94. Available from: http://linkinghub.elsevier.com/retrieve/pii/S0039606014000476

10. Chua WL, Mackey S, Ng EKC, Liaw SY. Front line nurses' experiences with deteriorating ward patients: A qualitative study. Int Nurs Rev. 2013;60(4):501–9.

11. Mochizuki K, Shintani R, Mori K, Sato T, Sakaguchi O, Takeshige K, et al. Importance of respiratory rate for the prediction of clinical deterioration after emergency department discharge: a single-center, case-control study. Acute Med Surg [Internet]. 2016;1–7. Available from: http://doi.wiley.com/10.1002/ams2.252

12. Brady PW, Goldenhar LM. A qualitative study examining the influences on situation awareness and the identification, mitigation and escalation of recognised patient risk. BMJ Qual Saf [Internet]. 2014 [cited 2017 May 9];23:153–61. Available from: http://dx.doi.org/10.1136/bmjqs-2012-001747

13. Shearer B, Marshall S, Buist MD, Finnigan M, Kitto S, Hore T, et al. What stops hospital clinical staff from following protocols? An analysis of the incidence and factors behind the failure of bedside clinical staff to activate the rapid response system in a multi-campus Australian metropolitan healthcare service. BMJ Qual Saf [Internet]. 2012;21(7):569–75. Available from: http://qualitysafety.bmj.com/lookup/doi/10.1136/bmjqs-2011-000692

14. Donohue LA, Endacott R. Track, trigger and teamwork: Communication of deterioration in acute medical and surgical wards Background. Intensive Crit Care Nurs [Internet]. 2010 [cited 2017 May 9];26. Available from: http://ac.els-cdn.com/S0964339709001074/1-s2.0-

For peer review only - http://bmjopen.bmj.com/site/about/guidelines.xhtml

S0964339709001074-main.pdf?_tid=42ebcfc6-34aa-11e7-a31b-00000aacb362&acdnat=1494329333_5d17d9cc08ce503e7b37debb709eaa99

15. Human Factors in Healthcare Patient Stories. 2013;

16. Greenhalgh T. HOW TO READ The basics of evidence based medicine [Internet]. Vol. 315, British Medical Journal. 2001. 596-9 p. Available from: http://www.pubmedcentral.nih.gov/articlerender.fcgi?artid=2127419&tool=pmcentrez&rendertype=abstract

17. Tacconelli E. Systematic reviews: CRD's guidance for undertaking reviews in health care [Internet]. Vol. 10, The Lancet Infectious Diseases. 2010. 226 p. Available from: http://linkinghub.elsevier.com/retrieve/pii/S1473309910700657

18. Gedda M. Traduction française des lignes directrices ENTREQ pour l'écriture et la lecture des synthèses de recherche qualitative. Kinesitherapie. 2015;15(157):55–8.

19. Barnett-Page E, Thomas J. Methods for the synthesis of qualitative research: A critical review. BMC Med Res Methodol. 2008;9(1).

For peer review only - http://bmjopen.bmj.com/site/about/guidelines.xhtml

Contributions

PW is the guarantor. JE was responsible for the overall design of the systematic review. JE and VW drafted the manuscript. TP and JE developed the search strategy. PW and JD provided systematic review and qualitative expertise. All authors read, provided feedback and approved the final manuscript.

Declarations

Funding

This systematic review protocol is funded by NIHR Biomedical Research Centre, based at Oxford University Hospitals Trust, Oxford and the Department of Health and Wellcome Trust through the Health Innovation Challenge Fund.

This publication presents independent research commissioned by the Health Innovation Challenge Fund (HICF-R9-524; WT-103703/Z/14/Z), a parallel funding partnership between the Department of Health and Wellcome Trust. The views expressed in this publication are those of the author(s) and not necessarily those of the Department of Health or Wellcome Trust. This work was also supported by the NIHR Biomedical Research Centre, Oxford.

Availability of data and materials

Not applicable

Consent for publication

Not applicable

Ethics approval and consent to participate

Not applicable

Competing interests

The authors declare that they have no competing interests.

For peer review only - http://bmjopen.bmj.com/site/about/guidelines.xhtml

Supplementary File 1 (Draft Search Strategy for MEDLINE)

1. HOSPITALIZATION/

2. TERTIARY CARE CENTERS/

3. (ward or wards).ab,ti.

4. (inhospital or inpatient* or "in hospital").ab,ti.

5. (hospitalised or hospitalized).ab,ti.

6. "general hospital".ab,ti.

7. "nurs* staff* ".ab,ti.

8. "in patient".ab,ti.

9. bedside.ab,ti.

10. outreach.ab,ti.

11. 1 or 2 or 3 or 4 or 5 or 6 or 7 or 8 or 9 or 10

12. FAILURE TO RESCUE,HEALTH CARE/

13. "fail* to rescue ".ab,ti.

14. VITAL SIGNS/

15. MONITORING,PHYSIOLOGIC/

16. "vital sign* ".ab,ti.

17. (track and trigger).ab,ti.

18."early warning".ab,ti.

19. "warning score* ".ab,ti.

20. "early sign* ".ab,ti.

21. "warning system* ".ab,ti.

22. (deteriorat* or escalat*).ab,ti.

23. triggering.ab,ti.

24. HOSPITAL RAPID RESPONSE TEAM/

25. "rapid response".ab,ti.

26. "critical care outreach".ab,ti.

27. 12 or 13 or 14 or 15 or 16 or 17 or 18 or 19 or 20 or 21 or 22 or 23 or 24 or 25 or 26

28. MEDICAL ERROR/

29. DELAYED DIAGNOSIS/

30. COMMUNICATION/

31. PATIENT CARE TEAMS/

For peer review only

32. PATIENT SAFETY/

33. ORGANIZATIONAL CULTURE/

34. LEADERSHIP/

35. "human factor* ".af.

36. "human error* ".af.

37. "clinical error* ".af.

38. "medical error* ".af.

39. "protocol adherence".af.

40. "protocol compliance".af.

41. "teamwork*".af.

42. communication.af.

43. ("socio cultural" or sociocultural).af.

44. "situation awareness".af.

45. "organisational culture".af.

46. "organizational culture".af.

47. "safety culture".af.

48. "patient safety".af.

49. leadership.af.

50. "root cause analysis".af.

51. 28 or 29 or 30 or 31 or 32 or 33 or 34 or 35 or 36 or 37 or 38 or 39 or 40 or 41 or 42 or 43 or 44 or 45 or 46 or 47 or 48 or 49 or 50

52. 11 and 27 and 51

For peer review only

PRISMA-P (Preferred Reporting Items for Systematic review and Meta-Analysis Protocols) 2015 checklist: recommended items to address in a systematic review protocol*

Section and topic	Item No	Checklist item	Page No
**ADMINISTRATIVE INFORMATION**			
Title:			
Identification	1a	Identify the report as a protocol of a systematic review	Pg 1 Line 3
Update	1b	If the protocol is for an update of a previous systematic review, identify as such	n/a
Registration	2	If registered, provide the name of the registry (such as PROSPERO) and registration number	Pg 2 Line 28<br>Pg 3 Lines 22-25
Authors:			
Contact	3a	Provide name, institutional affiliation, e-mail address of all protocol authors; provide physical mailing address of corresponding author	Pg 1 Lines 6-10
Contributions	3b	Describe contributions of protocol authors and identify the guarantor of the review	Pg 9 Lines 1-5
Amendments	4	If the protocol represents an amendment of a previously completed or published protocol, identify as such and list changes; otherwise, state plan for documenting important protocol amendments	n/a
Support:			
Sources	5a	Indicate sources of financial or other support for the review	Pg 1 Lines 13-16<br>Pg 9 Lines 6-10
Sponsor	5b	Provide name for the review funder and/or sponsor	Pg 1 Lines 13-16<br>Pg 9 Lines 6-10
Role of sponsor or funder	5c	Describe roles of funder(s), sponsor(s), and/or institution(s), if any, in developing the protocol	n/a
**INTRODUCTION**			
Rationale	6	Describe the rationale for the review in the context of what is already known	Pg 2 Lines 2-12<br>Pg 3 Lines 9-14
Objectives	7	Provide an explicit statement of the question(s) the review will address with reference to participants, interventions, comparators, and outcomes (PICO)	Pg 3 Lines 15-19
**METHODS**			
Eligibility criteria	8	Specify the study characteristics (such as PICO, study design, setting, time frame) and report characteristics (such as years considered, language, publication status) to be used as criteria for eligibility for the review	Pg 3 Lines 35-38<br>Pg 4 Lines 1-13

For peer review only

Information sources	9	Describe all intended information sources (such as electronic databases, contact with study authors, trial registers or other grey literature sources) with planned dates of coverage	Pg 3 Lines 26-31
Search strategy	10	Present draft of search strategy to be used for at least one electronic database, including planned limits, such that it could be repeated	Supplementary File 1
Study records:			
Data management	11a	Describe the mechanism(s) that will be used to manage records and data throughout the review	Pg 4 Lines 30-32<br>Pg 4 Lines 41-42<br>Pg 5 Line 1
Selection process	11b	State the process that will be used for selecting studies (such as two independent reviewers) through each phase of the review (that is, screening, eligibility and inclusion in meta-analysis)	Pg 4 Lines 30-39
Data collection process	11c	Describe planned method of extracting data from reports (such as piloting forms, done independently, in duplicate), any processes for obtaining and confirming data from investigators	Pg 4 Lines 40-42<br>Pg 5 Lines 1-2
Data items	12	List and define all variables for which data will be sought (such as PICO items, funding sources), any pre-planned data assumptions and simplifications	Pg 5 Lines 4-8 (inc table)
Outcomes and prioritization	13	List and define all outcomes for which data will be sought, including prioritization of main and additional outcomes, with rationale	Pg 3 Lines 15-19
Risk of bias in individual studies	14	Describe anticipated methods for assessing risk of bias of individual studies, including whether this will be done at the outcome or study level, or both; state how this information will be used in data synthesis	Pg 5 Lines 9-15
Data synthesis	15a	Describe criteria under which study data will be quantitatively synthesised	n/a
	15b	If data are appropriate for quantitative synthesis, describe planned summary measures, methods of handling data and methods of combining data from studies, including any planned exploration of consistency (such as I^2, Kendall's τ)	n/a
	15c	Describe any proposed additional analyses (such as sensitivity or subgroup analyses, meta-regression)	n/a
	15d	If quantitative synthesis is not appropriate, describe the type of summary planned	n/a
Meta-bias(es)	16	Specify any planned assessment of meta-bias(es) (such as publication bias across studies, selective reporting within studies)	Pg 5 Lines 14-15
Confidence in cumulative evidence	17	Describe how the strength of the body of evidence will be assessed (such as GRADE)	Pg 5 Lines 9-15

*** It is strongly recommended that this checklist be read in conjunction with the PRISMA-P Explanation and Elaboration (cite when available) for important clarification on the items. Amendments to a review protocol should be tracked and dated.** **The copyright for PRISMA-P (including checklist) is held by the PRISMA-P Group and is distributed under a Creative Commons Attribution Licence 4.0.**

From: Shamseer L, Moher D, Clarke M, Ghersi D, Liberati A, Petticrew M, Shekelle P, Stewart L, PRISMA-P Group. Preferred reporting items for systematic review and meta-analysis protocols (PRISMA-P) 2015: elaboration and explanation. BMJ. 2015 Jan 2;349(jan02 1):g7647.

For peer review only

BMJ Open

How human factors affect escalation of care: a protocol for a qualitative evidence synthesis of studies

Journal:	*BMJ Open*
Manuscript ID	bmjopen-2018-025969.R1
Article Type:	Protocol
Date Submitted by the Author:	09-Jan-2019
Complete List of Authors:	Ede, Jody; John Radcliffe Hospital, University of Oxford<br>Westgate, Verity ; University of Oxford, Nuffield Department of Clinical Neurosciences<br>Petrinic, Tatjana; University of Oxford Health Care Libraries, Cairns Library<br>Darbyshire, Julie; University of Oxford, NDCN<br>Watkinson, Peter; Oxford University Hospitals NHS Trust, Kadoorie Centre for Critical Care research and Education
**Primary Subject Heading**:	Qualitative research
Secondary Subject Heading:	Evidence based practice, Nursing, Intensive care, Research methods
Keywords:	Failure to rescue, Escalation of care, Human factors, Qualitative, Thematic syntheisis, QUALITATIVE RESEARCH

SCHOLARONE™
Manuscripts

Title Page

How human factors affect escalation of care: a protocol for a qualitative evidence synthesis of studies

Corresponding Author Jody Ede University of Oxford Jody.ede@ndcn.ox.ac.uk

Verity Westgate University of Oxford verity.westgate@ndcn.ox.ac.uk

Tatjana Petrinic Oxford University Hospital NHS Trust tatjana.petrinic@bodleian.ox.ac.uk

Julie Darbyshire University of Oxford Julie.darbyshire@ndcn.ox.ac.uk

Peter Watkinson Associate Professor University of Oxford peter.watkinson@ndcn.ox.ac.uk

"Supported by the NIHR Biomedical Research Centre, based at Oxford University Hospitals Trust, Oxford and the Wellcome Trust through the Health Innovation Challenge Fund. The views expressed are those of the author(s) and not necessarily those of the NHS, the NIHR or the Department of Health"

Word count: 1264

Keywords: Failure to rescue, escalation of care, human factors, qualitative, thematic, systematic

For peer review only

ABSTRACT (300)

Introduction

Failure to rescue is defined as mortality after complications during hospital care. Incidence ranges 10.9% - 13.3% and several national reports such as National Confidential Enquiry into Patient Outcomes and Death and National Institute of Clinical Excellence CG 50 highlight failure to rescue as a significant problem for safe patient care.

To avoid failure to rescue events, there must be successful escalation of care. Studies indicate that human factors such as situational awareness, team working, communication, and a culture promoting safety contribute to avoidance of failure to rescue events. Understanding human factors is essential to developing working-systems that mitigate barriers and facilitate prompt escalation of care. This qualitative evidence synthesis will identify and synthesise what is known about the human factors that affect escalation of care.

Methods and Analysis

We will search MEDLINE (Ovid), EMBASE (Ovid), and CINAHL for studies describing human factors affecting both failure to rescue and/or care escalation. A search strategy was developed by two researchers and a medical librarian. Only studies exploring in-hospital (ward) populations using qualitative data collection methods will be included. Screening will be conducted by two researchers from different professional backgrounds. We are likely to undertake a thematic synthesis, using the Thomas and Harden framework. Selected studies will be assessed for quality, rigor and limitations. Two researchers will extract and thematically synthesise codes using a piloted data extraction tool to develop analytical themes.

Ethics and dissemination

The qualitative evidence synthesis will use available published literature and no ethical approval is required. This synthesis will be limited by the quality of studies, rigor and reproducibility of study findings. This publication will synthesise what is known about human factors and escalation of care, highlighting gaps within the literature. Results will be published in a peer-reviewed journal, publicised at conferences and on social media.

TRIAL REGISTRATION

PROSPERO: (CRD42018104745)

ARTICLE SUMMARY

Strengths and limitations of this study

- Failure to rescue is a common problem in healthcare with significant effects on patient mortality
- For failure to rescue to be avoided, an escalation of care needs to occur. The efficacy of this can be positively or negatively affected by human factors
- This protocol ensures a comprehensive and unbiased search and analysis of qualitative studies exploring this phenomenon using best practice guidelines
- The results of this review will identify strengths and weaknesses of the literature in this area
- This review will highlight potential research direction for future studies and will address some of the weaknesses identified in published research projects

For peer review only

For peer review only - http://bmjopen.bmj.com/site/about/guidelines.xhtml

INTRODUCTION

Failure to rescue is defined as the mortality rate of patients who suffer complications in hospital (1). The incidence of failure to rescue events varies between hospitals but has been estimated as 10.9% in high-volume hospitals and 13.3% in low-volume hospitals (2). A proportion of patient deaths (32%) reported to the National Patient Safety Agency (NSPA) had failures surrounding diagnostic errors and deteriorations which were not adequately recognised (3). Failure to recognise the need to rescue patients by providing timely escalation of care is a finding in several national reports such as National Confidential Enquiry into Patient Outcomes and Death (NCEPOD) (4–7) and NICE CG 50 (8).

For ‘failure to rescue’ to be avoided, bedside clinical staff must usually initiate successful escalation of care (9). This staged process requires detection of deterioration, communication about deterioration, and medical actions following senior review (4). Many factors affect this process such as situational awareness, team working, communication, safety culture and leadership (4,10–14). Understanding these human factors is essential to developing working systems that mitigate barriers and facilitate prompt escalation of care.

The aim of this qualitative evidence synthesis is to map the human factors which affect escalation of care in the acute hospital setting. It will summarise what is currently understood about the role human factors play in the delivery of good clinical care. Secondly, it will identify gaps in the current literature and establish strengths and weaknesses of research conducted to date. This will produce an evidence base from which escalation of care theory could be developed. We will also identify potential areas for further research in human factors and the escalation of care process.

Methods and Analysis

Registration

This protocol adheres to the requirements of Preferred reporting items for systematic review and meta-analysis protocols (PRISMA-P). The protocol was registered with PROSPERO (ref: CRD42018104745)

Information sources

Literature search strategies will be developed using Medical Subject Headings (MeSH) and text words related to the human factors involved in the escalation of care for deteriorating patients.

The following databases will be searched: MEDLINE (Ovid), EMBASE (Ovid), and CINAHL.

Reference lists of eligible studies and relevant reviews will be explored to identify further eligible studies.

Search strategy

A draft of the search strategy was developed by three of the authors (JE, VW and TP). The proposed search strategy is shown in the online Supplementary File 1.

Inclusion Criteria

For peer review only - http://bmjopen.bmj.com/site/about/guidelines.xhtml

Types of studies

This qualitative evidence synthesis will include qualitative studies which report primary data. Qualitative studies are defined as those using qualitative data collection and analysis methods. These can include, but are not limited to, ethnography, interviews, focus groups and human factors methods. Data analysis is likely (but not limited) to include thematic analysis, grounded theory, and/or discourse analysis. We will also include grey literature. All studies meeting inclusion criteria will be included and reviewed.

Study focus

Studies must report primary data and describe human factors affecting failure to rescue and escalation of care. Failure to rescue is defined as patient mortality following complications (1) and escalation of care is a staged process where patients are identified as 'deteriorating', and that deterioration is then communicated followed by senior review and medical intervention where necessary (4). We will include any qualitative study which explores the perspective of patients or clinical staff (adults or paediatric) and the human factors which affect the escalation of care process. We are defining human factors as any barrier or facilitator that affects teamwork, tasks, equipment, workspace, culture, or organisation (15).

Setting

The study setting is in-hospital, ward care.

Exclusion criteria

Types of studies

We will exclude systematic reviews, editorials, letters, practice guidelines and abstract-only reports. We will also exclude protocols without study data.

Phenomenon of interest

We are only interested in real-life scenarios where human factors effects can be studied in the patient environment. Simulation based studies will be excluded.

Setting

We will exclude studies carried out in the Emergency Department, Critical Care (including the Intensive Care Unit and Coronary care) or Maternity. These are specialised areas which makes it challenging to generalise to the ward environment any 'escalation of care' practices identified in these areas. We will also exclude studies set in palliative care.

Time-frame

No time limitations will be applied

Language

Non-English papers will be excluded.

Study selection

Reference lists from all databases will be entered into Covidence software (Covidence systematic review software, Veritas Health Innovation, Melbourne, Australia. Available at www.covidence.org). Papers will be de-duplicated. Two authors will independently screen titles and abstracts of identified papers against the inclusion and exclusion criteria. They will not be blinded to journal titles, study authors or institutions. If there is disagreement or uncertainty regarding eligibility, the full-text will be reviewed. We will retrieve full-text for all articles not excluded by the initial screening. Two authors will independently assess these papers against the inclusion and exclusion criteria outlined above. We will resolve disagreements about eligibility by discussion between the screening researchers or a third party. We will record the reason for excluding studies.

For peer review only - http://bmjopen.bmj.com/site/about/guidelines.xhtml

Data extraction

Data extraction tools will be developed and piloted before the review takes place. Extracted data will be entered into Excel (Microsoft Office 2016). Initial coding will be documented with NVivo [NVivo qualitative data analysis Software; QSR International Pty Ltd. Version 10, 2014]. Two reviewers will independently extract a selection data from the texts to ensure validity of results. Any discrepancies within the data collection phase will be resolved by discussion between reviewers or a third party.

Data items extracted

We will extract the following data from each included publication (refer to Table. 1 for full data details). The data extraction method has been piloted with a sample selection of papers and valid data have been obtained.

Table 1- Anticipated data to be extracted

Study Characteristics	Patient/Participant demographics	Study setting	Themes	Rigor
• Author<br>• Date of study<br>• Study Type<br>• Methodology<br>• Country of study<br>• Data collection methods<br>• Journal<br>• Data analyses	• Age<br>• Patient group<br>• In-patient characterisation	• Level of care<br>• Hospital Type<br>• Education	• Codes	• Strengths<br>• Weaknesses<br>• Reporting guidelines used

Quality Assessment

The CASP qualitative checklist (Critical Appraisal Skills Programme) will be used to assess credibility, transferability, dependability and confirmability. This checklist is an extensive and comprehensive tool commonly used in qualitative study assessment (16,17). As part of the CASP assessment the authors will explore the potential for reporting bias within the studies and biases will be reported in studies' limitations. We will also apply the Confidence in the Evidence from Reviews of Qualitative research (GRADE-CERQual) criteria to judge studies (18). Two researchers will discuss each study and a consensus will be reached to include or exclude.

Data Analysis

This review aims to explore relevant theory and map barriers and facilitators to escalation of care for which thematic synthesis is well suited (17). We are likely to undertake a thematic synthesis, using the Thomas and Harden framework (19). This framework supports data extraction from anywhere within the paper, and is not confined to the results alone. The three stages of the framework are: coding findings from included studies, categorisation of codes into descriptive themes, and categorisation of descriptive themes into analytical themes (19). Stage one involves line by line coding of data, where each sentence is allocated a code. Stage two involves categorising each coded sentence into descriptive, broader themes. The final stage involves generating analytical themes, or 'going beyond' the findings of the initial study, which relate to the fixed or emerging research question. Whilst we have been explicit at this point as to the anticipated framework, it is also

For peer review only - http://bmjopen.bmj.com/site/about/guidelines.xhtml

For peer review only

justifiable for this to change once the search has been conducted (20). NVivo software will be used to code the original text from the papers. Using this software will facilitate analysis for this evidence synthesis and will be used to record decisions (by audit trail) of coding. Codes relating to human factors and escalation of care will be identified from anywhere within the papers, and tables will be used to record descriptive and analytical themes. Key codes, descriptive themes and analytical themes will be presented in the results. We will use the enhancing transparency in reporting the synthesis of qualitative research (ENTREQ) guidelines to report findings (21).

Patient and Public Involvement (PPI)

A patient representative has read and provided feedback on the protocol. As a result, some points have been clarified and medical "jargon" removed.

Ethics and dissemination

The proposed evidence synthesis will use published literature and therefore no ethical approval is required. This publication will be limited by the quality of studies available and the rigor and reproducibility of study findings. Original studies included in the review could themselves be limited and it may be difficult to assess the researcher involvement and their individual bias. The two researchers carrying out screening for this review come from different professional backgrounds, limiting interpretation bias when assessing the studies for inclusion. A recognised assessment tool will be used to determine study quality. Using NVivo to code studies will aid transparency and demonstrate a clear strategy for theme identification. An audit trail kept throughout the process, will detail decisions made and methodological steps taken.

The results from this review will be published and made freely available. A number of social media techniques (including Twitter, Facebook, and our research group website) will be used to promote the protocol, final paper and results. We will also aim to attend at least one conference to present findings from this work.

For peer review only - http://bmjopen.bmj.com/site/about/guidelines.xhtml

References

1. Ghaferi AA, Osborne NH, Birkmeyer JD, Dimick JB. Hospital characteristics associated with failure to rescue from complications after pancreatectomy. J Am Coll Surg [Internet]. Elsevier Inc.; 2010;211(3):325–30. Available from: http://dx.doi.org/10.1016/j.jamcollsurg.2010.04.025

2. Ghaferi AA, Birkmeyer JD, Dimick JB. Hospital volume and failure to rescue with high-risk surgery. Med Care. 2011;49(12):1076–81.

3. NHS Improvement. The adult patient who is deteriorating: sharing learning from literature, incident reports and root cause analysis investigations. 2016;(July):23. Available from: https://improvement.nhs.uk/documents/176/Deterioration_in_adults_report_7july.pdf

4. Findlay G, Shotton H, Mason K. Time to Intervene? Natl Confid Enq into Patient Oucome Death [Internet]. 2012;1–125. Available from: http://www.ncepod.org.uk/2012report1/downloads/CAP_fullreport.pdf

5. Fox P. An acute problem. Nurs Elder. 2005;3(4):33.

6. Harper SJ. NCEPOD: Caring to the end? J Intensive Care Soc. 2010;11(2):88–9.

7. National Confidential Enquiry into Patient Outcome and Death. Emergency Admissions: A jouney in the right direction? 2007; Available from: http://www.ncepod.org.uk/2007ea.htm

8. NICE. Acutely ill adults in hospital: recognising Acutely ill adults in hospital: recognising and responding to deterior and responding to deterioration (CG50). 2007;(July). Available from: www.nice.org.uk/guidance/cg50

9. Johnston M, Arora S, King D, Stroman L, Darzi A. Escalation of care and failure to rescue: A multicenter, multiprofessional qualitative study. Surgery [Internet]. 2014 Jun [cited 2017 Aug 7];155(6):989–94. Available from:

For peer review only

For peer review only - http://bmjopen.bmj.com/site/about/guidelines.xhtml

http://linkinghub.elsevier.com/retrieve/pii/S0039606014000476

10. Chua WL, Mackey S, Ng EKC, Liaw SY. Front line nurses' experiences with deteriorating ward patients: A qualitative study. Int Nurs Rev. 2013;60(4):501–9.

11. Mochizuki K, Shintani R, Mori K, Sato T, Sakaguchi O, Takeshige K, et al. Importance of respiratory rate for the prediction of clinical deterioration after emergency department discharge: a single-center, case-control study. Acute Med Surg [Internet]. 2016;1–7. Available from: http://doi.wiley.com/10.1002/ams2.252

12. Brady PW, Goldenhar LM. A qualitative study examining the influences on situation awareness and the identification, mitigation and escalation of recognised patient risk. BMJ Qual Saf [Internet]. 2014 [cited 2017 May 9];23:153–61. Available from: http://dx.doi.org/10.1136/bmjqs-2012-001747

13. Shearer B, Marshall S, Buist MD, Finnigan M, Kitto S, Hore T, et al. What stops hospital clinical staff from following protocols? An analysis of the incidence and factors behind the failure of bedside clinical staff to activate the rapid response system in a multi-campus Australian metropolitan healthcare service. BMJ Qual Saf [Internet]. 2012;21(7):569–75. Available from: http://qualitysafety.bmj.com/lookup/doi/10.1136/bmjqs-2011-000692

14. Donohue LA, Endacott R. Track, trigger and teamwork: Communication of deterioration in acute medical and surgical wards Background. Intensive Crit Care Nurs [Internet]. 2010 [cited 2017 May 9];26. Available from: http://ac.els-cdn.com/S0964339709001074/1-s2.0-S0964339709001074-main.pdf?_tid=42ebcfc6-34aa-11e7-a31b-00000aacb362&acdnat=1494329333_5d17d9cc08ce503e7b37debb709eaa99

15. Human Factors in Healthcare Patient Stories. 2013;

16. Greenhalgh T. HOW TO READ The basics of evidence based medicine [Internet]. Vol. 315, British Medical Journal. 2001. 596-9 p. Available from:

For peer review only - http://bmjopen.bmj.com/site/about/guidelines.xhtml

http://www.pubmedcentral.nih.gov/articlerender.fcgi?artid=2127419&tool=pmcentrez&rendertype=abstract

17. Tacconelli E. Systematic reviews: CRD's guidance for undertaking reviews in health care [Internet]. Vol. 10, The Lancet Infectious Diseases. 2010. 226 p. Available from: http://linkinghub.elsevier.com/retrieve/pii/S1473309910700657

18. Lewin S, Booth A, Glenton C, Munthe-Kaas H, Rashidian A, Wainwright M, et al. Applying GRADE-CERQual to qualitative evidence synthesis findings: introduction to the series. Implement Sci [Internet]. 2018 [cited 2018 Dec 17];13(1). Available from: http://creativecommons.org/publicdomain/zero/1.0/

19. Barnett-Page E, Thomas J. Methods for the synthesis of qualitative research: A critical review. BMC Med Res Methodol. 2008;9(1).

20. Dixon-Woods M. Using framework-based synthesis for conducting reviews of qualitative studies. BMC Med. 2011;9:9–10.

21. Enhancing transparency in reporting the synthesis of qualitative research: ENTREQ | The EQUATOR Network [Internet]. [cited 2018 Dec 14]. Available from: http://www.equator-network.org/reporting-guidelines/entreq/

For peer review only

For peer review only - http://bmjopen.bmj.com/site/about/guidelines.xhtml

For peer review only

For peer review only - http://bmjopen.bmj.com/site/about/guidelines.xhtml

Contributions

PW is the guarantor. JE was responsible for the overall design of the QES. JE and VW drafted the manuscript. TP and JE developed the search strategy. PW and JD provided QES and qualitative expertise. All authors read, provided feedback and approved the final manuscript. We would like to thank patient representative, TD for his contribution to this work.

Declarations

Funding

This QES protocol is funded by NIHR Biomedical Research Centre, based at Oxford University Hospitals Trust, Oxford and the Department of Health and Wellcome Trust through the Health Innovation Challenge Fund.

This publication presents independent research commissioned by the Health Innovation Challenge Fund (HICF-R9-524; WT-103703/Z/14/Z), a parallel funding partnership between the Department of Health and Wellcome Trust. The views expressed in this publication are those of the author(s) and not necessarily those of the Department of Health or Wellcome Trust. This work was also supported by the NIHR Biomedical Research Centre, Oxford.

Availability of data and materials

Not applicable

Consent for publication

Not applicable

Ethics approval and consent to participate

Not applicable

Competing interests

The authors declare that they have no competing interests.

For peer review only - http://bmjopen.bmj.com/site/about/guidelines.xhtml

Supplementary File 1 (Draft Search Strategy for MEDLINE)

1. HOSPITALIZATION/

2. TERTIARY CARE CENTERS/

3. (ward or wards).ab,ti.

4. (inhospital or inpatient* or "in hospital").ab,ti.

5. (hospitalised or hospitalized).ab,ti.

6. "general hospital".ab,ti.

7. "nurs* staff* ".ab,ti.

8. "in patient".ab,ti.

9. bedside.ab,ti.

10. outreach.ab,ti.

11. 1 or 2 or 3 or 4 or 5 or 6 or 7 or 8 or 9 or 10

12. FAILURE TO RESCUE,HEALTH CARE/

13. "fail* to rescue ".ab,ti.

14. VITAL SIGNS/

15. MONITORING,PHYSIOLOGIC/

16. "vital sign* ".ab,ti.

17. (track and trigger).ab,ti.

18."early warning".ab,ti.

19. "warning score* ".ab,ti.

20. "early sign* ".ab,ti.

21. "warning system* ".ab,ti.

22. (deteriorat* or escalat*).ab,ti.

23. triggering.ab,ti.

24. HOSPITAL RAPID RESPONSE TEAM/

25. "rapid response".ab,ti.

26. "critical care outreach".ab,ti.

27. 12 or 13 or 14 or 15 or 16 or 17 or 18 or 19 or 20 or 21 or 22 or 23 or 24 or 25 or 26

28. MEDICAL ERROR/

29. DELAYED DIAGNOSIS/

30. COMMUNICATION/

31. PATIENT CARE TEAMS/

For peer review only

32. PATIENT SAFETY/

33. ORGANIZATIONAL CULTURE/

34. LEADERSHIP/

35. "human factor* ".af.

36. "human error* ".af.

37. "clinical error* ".af.

38. "medical error* ".af.

39. "protocol adherence".af.

40. "protocol compliance".af.

41. "teamwork*".af.

42. communication.af.

43. ("socio cultural" or sociocultural).af.

44. "situation awareness".af.

45. "organisational culture".af.

46. "organizational culture".af.

47. "safety culture".af.

48. "patient safety".af.

49. leadership.af.

50. "root cause analysis".af.

51. 28 or 29 or 30 or 31 or 32 or 33 or 34 or 35 or 36 or 37 or 38 or 39 or 40 or 41 or 42 or 43 or 44 or 45 or 46 or 47 or 48 or 49 or 50

52. 11 and 27 and 51

For peer review only

PRISMA-P (Preferred Reporting Items for Systematic review and Meta-Analysis Protocols) 2015 checklist: recommended items to address in a systematic review protocol*

Section and topic	Item No	Checklist item	Page No
**ADMINISTRATIVE INFORMATION**			
Title:			
Identification	1a	Identify the report as a protocol of a systematic review	Pg 1 Line 3
Update	1b	If the protocol is for an update of a previous systematic review, identify as such	n/a
Registration	2	If registered, provide the name of the registry (such as PROSPERO) and registration number	Pg 2 Line 29<br>Pg 3 Lines 25-26
Authors:			
Contact	3a	Provide name, institutional affiliation, e-mail address of all protocol authors; provide physical mailing address of corresponding author	Pg 1 Lines 6-10
Contributions	3b	Describe contributions of protocol authors and identify the guarantor of the review	Pg 10 Lines 1-4
Amendments	4	If the protocol represents an amendment of a previously completed or published protocol, identify as such and list changes; otherwise, state plan for documenting important protocol amendments	n/a
Support:			
Sources	5a	Indicate sources of financial or other support for the review	Pg 1 Lines 13-16<br>Pg 9 Lines 6-14
Sponsor	5b	Provide name for the review funder and/or sponsor	Pg 1 Lines 13-16<br>Pg 9 Lines 6-10
Role of sponsor or funder	5c	Describe roles of funder(s), sponsor(s), and/or institution(s), if any, in developing the protocol	n/a
**INTRODUCTION**			
Rationale	6	Describe the rationale for the review in the context of what is already known	Pg 2 Lines 2-12<br>Pg 3 Lines 1-14
Objectives	7	Provide an explicit statement of the question(s) the review will address with reference to participants, interventions, comparators, and outcomes (PICO)	Pg 3 Lines 15-20
**METHODS**			
Eligibility criteria	8	Specify the study characteristics (such as PICO, study design, setting, time frame) and report characteristics (such as years considered, language, publication status) to be used as criteria for eligibility for the review	Pg 3 Lines 34-36<br>Pg 4 Lines 1-18

For peer review only

Information sources	9	Describe all intended information sources (such as electronic databases, contact with study authors, trial registers or other grey literature sources) with planned dates of coverage	Pg 3 Lines 27-32
Search strategy	10	Present draft of search strategy to be used for at least one electronic database, including planned limits, such that it could be repeated	Supplementary File 1
Study records:			
Data management	11a	Describe the mechanism(s) that will be used to manage records and data throughout the review	Pg 4 Lines 36-38<br>Pg 5 Lines 2-6
Selection process	11b	State the process that will be used for selecting studies (such as two independent reviewers) through each phase of the review (that is, screening, eligibility and inclusion in meta-analysis)	Pg 4 Lines 38-44
Data collection process	11c	Describe planned method of extracting data from reports (such as piloting forms, done independently, in duplicate), any processes for obtaining and confirming data from investigators	Pg 5 Lines 2-6
Data items	12	List and define all variables for which data will be sought (such as PICO items, funding sources), any pre-planned data assumptions and simplifications	Pg 4 Lines 7-10(inc table)
Outcomes and prioritization	13	List and define all outcomes for which data will be sought, including prioritization of main and additional outcomes, with rationale	Pg 4 Lines 9-10
Risk of bias in individual studies	14	Describe anticipated methods for assessing risk of bias of individual studies, including whether this will be done at the outcome or study level, or both; state how this information will be used in data synthesis	Pg 5 Lines 13-20
Data synthesis	15a	Describe criteria under which study data will be quantitatively synthesised	n/a
	15b	If data are appropriate for quantitative synthesis, describe planned summary measures, methods of handling data and methods of combining data from studies, including any planned exploration of consistency (such as I^2, Kendall's τ)	n/a
	15c	Describe any proposed additional analyses (such as sensitivity or subgroup analyses, meta-regression)	n/a
	15d	If quantitative synthesis is not appropriate, describe the type of summary planned	n/a
Meta-bias(es)	16	Specify any planned assessment of meta-bias(es) (such as publication bias across studies, selective reporting within studies)	Pg 5 Lines 13-20
Confidence in cumulative evidence	17	Describe how the strength of the body of evidence will be assessed (such as GRADE)	Pg 5 Lines 19-20

*** It is strongly recommended that this checklist be read in conjunction with the PRISMA-P Explanation and Elaboration (cite when available) for important clarification on the items. Amendments to a review protocol should be tracked and dated.** **The copyright for PRISMA-P (including checklist) is held by the PRISMA-P Group and is distributed under a Creative Commons Attribution Licence 4.0.**

From: Shamseer L, Moher D, Clarke M, Ghersi D, Liberati A, Petticrew M, Shekelle P, Stewart L, PRISMA-P Group. Preferred reporting items for systematic review and meta-analysis protocols (PRISMA-P) 2015: elaboration and explanation. BMJ. 2015 Jan 2;349(jan02 1):g7647.

For peer review only

BMJ Open

How human factors affect escalation of care: a protocol for a qualitative evidence synthesis of studies

Journal:	*BMJ Open*
Manuscript ID	bmjopen-2018-025969.R2
Article Type:	Protocol
Date Submitted by the Author:	05-Feb-2019
Complete List of Authors:	Ede, Jody; John Radcliffe Hospital, University of Oxford<br>Westgate, Verity ; University of Oxford, Nuffield Department of Clinical Neurosciences<br>Petrinic, Tatjana; University of Oxford Health Care Libraries, Cairns Library<br>Darbyshire, Julie; University of Oxford, NDCN<br>Watkinson, Peter; Oxford University Hospitals NHS Trust, Kadoorie Centre for Critical Care research and Education
**Primary Subject Heading**:	Qualitative research
Secondary Subject Heading:	Evidence based practice, Nursing, Intensive care, Research methods
Keywords:	Failure to rescue, Escalation of care, Human factors, Qualitative, Thematic syntheisis, QUALITATIVE RESEARCH

SCHOLARONE™
Manuscripts

Title Page

How human factors affect escalation of care: a protocol for a qualitative evidence synthesis of studies

Corresponding Author Jody Ede University of Oxford Jody.ede@ndcn.ox.ac.uk

Verity Westgate University of Oxford verity.westgate@ndcn.ox.ac.uk

Tatjana Petrinic Oxford University Hospital NHS Trust tatjana.petrinic@bodleian.ox.ac.uk

Julie Darbyshire University of Oxford Julie.darbyshire@ndcn.ox.ac.uk

Peter Watkinson Associate Professor University of Oxford peter.watkinson@ndcn.ox.ac.uk

"Supported by the NIHR Biomedical Research Centre, based at Oxford University Hospitals Trust, Oxford and the Wellcome Trust through the Health Innovation Challenge Fund. The views expressed are those of the author(s) and not necessarily those of the NHS, the NIHR or the Department of Health"

Word count: 1264

Keywords: Failure to rescue, escalation of care, human factors, qualitative, thematic, systematic

For peer review only

ABSTRACT

Introduction

Failure to rescue is defined as mortality after complications during hospital care. Incidence ranges 10.9% - 13.3% and several national reports such as National Confidential Enquiry into Patient Outcomes and Death and National Institute of Clinical Excellence CG 50 highlight failure to rescue as a significant problem for safe patient care.

To avoid failure to rescue events, there must be successful escalation of care. Studies indicate that human factors such as situational awareness, team working, communication, and a culture promoting safety contribute to avoidance of failure to rescue events. Understanding human factors is essential to developing work-systems that mitigate barriers and facilitate prompt escalation of care. This qualitative evidence synthesis will identify and synthesise what is known about the human factors that affect escalation of care.

Methods and Analysis

We will search MEDLINE (Ovid), EMBASE (Ovid), and CINAHL, between database inception and 2018, for studies describing human factors affecting failure to rescue and/or care escalation. A search strategy was developed by two researchers and a medical librarian. Only studies exploring in-hospital (ward) populations using qualitative data collection methods will be included. Screening will be conducted by two researchers. We are likely to undertake a thematic synthesis, using the Thomas and Harden framework. Selected studies will be assessed for quality, rigor and limitations. Two researchers will extract and thematically synthesise codes using a piloted data extraction tool to develop analytical themes.

Ethics and dissemination

The qualitative evidence synthesis will use available published literature and no ethical approval is required. This synthesis will be limited by the quality of studies, rigor and reproducibility of study findings. Results will be published in a peer-reviewed journal, publicised at conferences and on social media.

TRIAL REGISTRATION

PROSPERO: (CRD42018104745)

ARTICLE SUMMARY

Strengths and limitations of this study

- Failure to rescue is a common problem in healthcare with significant effects on patient mortality
- For failure to rescue to be avoided, an escalation of care needs to occur. The efficacy of this can be positively or negatively affected by human factors
- This protocol ensures a comprehensive and unbiased search and analysis of qualitative studies exploring this phenomenon using best practice guidelines
- The results of this review will identify strengths and weaknesses of the literature in this area
- This review will highlight potential research direction for future studies and will address some of the weaknesses identified in published research projects

For peer review only - http://bmjopen.bmj.com/site/about/guidelines.xhtml

INTRODUCTION

Failure to rescue is defined as the mortality rate of patients who suffer complications in hospital (1). The incidence of failure to rescue events varies between hospitals but has been estimated as 10.9% in high-volume hospitals and 13.3% in low-volume hospitals (2). A proportion of patient deaths (32%) reported to the National Patient Safety Agency (NSPA) had failures surrounding diagnostic errors and deteriorations which were not adequately recognised (3). Failure to recognise the need to rescue patients by providing timely escalation of care is a finding in several national reports such as National Confidential Enquiry into Patient Outcomes and Death (NCEPOD) (4–7) and NICE CG 50 (8).

For 'failure to rescue' to be avoided, bedside clinical staff must usually initiate successful escalation of care (9). This staged process requires detection of deterioration, communication about deterioration, and medical actions following senior review (4). Many factors affect this process such as situational awareness, team working, communication, safety culture and leadership (4,10–14). Understanding these human factors is essential to developing working systems that mitigate barriers and facilitate prompt escalation of care.

The aim of this qualitative evidence synthesis is to map the human factors which affect escalation of care in the acute hospital setting. It will summarise what is currently understood about the role human factors play in the delivery of clinical care. Secondly, it will identify gaps in the current literature and establish strengths and weaknesses of research conducted to date. This will produce an evidence base from which escalation of care theory could be developed. We will also identify potential areas for further research in human factors and the escalation of care process.

Methods and Analysis

Registration

This protocol adheres to the requirements of Preferred reporting items for systematic review and meta-analysis protocols (PRISMA-P). The protocol was registered with PROSPERO (ref: CRD42018104745)

Information sources

Literature search strategies will be developed using Medical Subject Headings (MeSH) and text words related to the human factors involved in the escalation of care for deteriorating patients.

The following databases will be searched: MEDLINE (Ovid), EMBASE (Ovid), and CINAHL. Dates searched will be from database inception to January 2018.

Reference lists of eligible studies and relevant reviews will be explored to identify further eligible studies.

Search strategy

A draft of the search strategy was developed by three of the authors (JE, VW and TP). The proposed search strategy is shown in the online Supplementary File 1.

Inclusion Criteria

For peer review only - http://bmjopen.bmj.com/site/about/guidelines.xhtml

Types of studies

This qualitative evidence synthesis will include qualitative studies which report primary data. Qualitative studies are defined as those using qualitative data collection and analysis methods. These can include, but are not limited to, ethnography, interviews, focus groups and human factors methods. Data analysis is likely (but not limited) to include thematic analysis, grounded theory, and/or discourse analysis. We will also include grey literature. All studies meeting inclusion criteria will be included and reviewed.

Study focus

Studies must report primary data and describe human factors affecting failure to rescue and escalation of care. Failure to rescue is defined as patient mortality following complications (1) and escalation of care is a staged process where patients are identified as 'deteriorating', and that deterioration is then communicated followed by senior review and medical intervention where necessary (4). We will include any qualitative study which explores the perspective of patients or clinical staff (adults or paediatric) and the human factors which affect the escalation of care process. We are defining human factors as any barrier or facilitator that affects teamwork, tasks, equipment, workspace, culture, or organisation (15).

Setting

The study setting is in-hospital, ward care.

Exclusion criteria

Types of studies

We will exclude systematic reviews, editorials, letters, practice guidelines and abstract-only reports. We will also exclude protocols without study data.

Phenomenon of interest

We are only interested in real-life scenarios where human factors effects can be studied in the patient environment. Simulation based studies will be excluded.

Setting

We will exclude studies carried out in the Emergency Department, Critical Care (including the Intensive Care Unit and Coronary care) or Maternity. These are specialised areas which makes it challenging to generalise to the ward environment any 'escalation of care' practices identified in these areas. We will also exclude studies set in palliative care.

Time-frame

No time limitations will be applied

Language

Non-English papers will be excluded.

Study selection

Reference lists from all databases will be entered into Covidence software (Covidence systematic review software, Veritas Health Innovation, Melbourne, Australia. Available at www.covidence.org). Papers will be de-duplicated. Two authors will independently screen titles and abstracts of identified papers against the inclusion and exclusion criteria. They will not be blinded to journal titles, study authors or institutions. If there is disagreement or uncertainty regarding eligibility, the full-text will be reviewed. We will retrieve full-text for all articles not excluded by the initial screening. Two authors will independently assess these papers against the inclusion and exclusion criteria outlined above. Papers which inclusion is uncertain, will be fully reviewed for synthesis suitability. We will

For peer review only - http://bmjopen.bmj.com/site/about/guidelines.xhtml

resolve disagreements about eligibility by discussion between the screening researchers or a third party. We will record the reason for excluding studies.

Data extraction

Data extraction tools will be developed and piloted before the review takes place. Extracted data will be entered into Excel (Microsoft Office 2016). Initial coding will be documented with NVivo [NVivo qualitative data analysis Software; QSR International Pty Ltd. Version 10, 2014]. Two reviewers will independently extract a selection data from the texts to ensure validity of results. Any discrepancies within the data collection phase will be resolved by discussion between reviewers or a third party.

Data items extracted

We will extract the following data from each included publication (refer to Table. 1 for full data details). The data extraction method has been piloted with a sample selection of papers and valid data have been obtained.

Table 1- Anticipated data to be extracted

Study Characteristics	Patient/Participant demographics	Study setting	Themes	Rigor
• Author<br>• Date of study<br>• Study Type<br>• Methodology<br>• Country of study<br>• Data collection methods<br>• Journal<br>• Data analyses	• Age<br>• Patient group<br>• In-patient characterisation	• Level of care<br>• Hospital Type<br>• Education	• Codes	• Strengths<br>• Weaknesses<br>• Reporting guidelines used

Quality Assessment

The CASP qualitative checklist (Critical Appraisal Skills Programme) will be used to assess credibility, transferability, dependability and confirmability. This checklist is an extensive and comprehensive tool commonly used in qualitative study assessment (16,17). As part of the CASP assessment the authors will explore the potential for reporting bias within the studies and biases will be reported in studies' limitations. Two researchers will discuss each study and a consensus will be reached to include or exclude.

Assessment of confidence in synthesised findings

We will apply the Confidence in the Evidence from Reviews of Qualitative research (GRADE-CERQual) criteria to judge confidence in synthesised findings (18). We will apply the CERQual criteria to each study finding, assessing for methodological limitations, relevance, coherence and adequacy of data. This method will generate a Summary of Qualitative of Study Findings (SoQF) table, providing a transparent method with which to assess included studies and results (18).

Data Analysis

For peer review only

For peer review only - http://bmjopen.bmj.com/site/about/guidelines.xhtml

This review aims to explore relevant theory and map barriers and facilitators to escalation of care for which thematic synthesis is well suited (17). We are likely to undertake a thematic synthesis, using the Thomas and Harden framework (19). This framework supports data extraction from anywhere within the paper, and is not confined to the results alone. The three stages of the framework are: coding findings from included studies, categorisation of codes into descriptive themes, and categorisation of descriptive themes into analytical themes (19). Stage one involves line by line coding of data, where each sentence is allocated a code. Stage two involves categorising each coded sentence into descriptive, broader themes. The final stage involves generating analytical themes, or 'going beyond' the findings of the initial study, which relate to the fixed or emerging research question. Whilst we have been explicit at this point as to the anticipated framework, it is also justifiable for this to change once the search has been conducted (20).

NVivo software will be used to code the original text from papers. Using this software will facilitate analysis for this evidence synthesis and will be used to record decisions (by audit trail) of coding. Codes relating to human factors and escalation of care will be identified from anywhere within the papers, and tables will be used to record descriptive and analytical themes. Key codes, descriptive themes and analytical themes will be presented in the results. We will use the enhancing transparency in reporting the synthesis of qualitative research (ENTREQ) guidelines to report findings (21).

Patient and Public Involvement (PPI)

A patient representative (TD) has read and provided feedback on the protocol. As a result, some points have been clarified and medical "jargon" removed.

Ethics and dissemination

The proposed evidence synthesis will use published literature and therefore no ethical approval is required. This publication will be limited by the quality of studies available and the rigor and reproducibility of study findings. Original studies included in the review could themselves be limited and it may be difficult to assess the researcher involvement and their individual bias. The two researchers carrying out screening for this review come from different professional backgrounds, limiting interpretation bias when assessing the studies for inclusion. A recognised assessment tool will be used to determine study quality. Using NVivo to code studies will aid transparency and demonstrate a clear strategy for theme identification. An audit trail kept throughout the process, will detail decisions made and methodological steps taken.

The results from this review will be published and made freely available. A number of social media techniques (including Twitter, Facebook, and our research group website) will be used to promote the protocol, final paper and results. We will also aim to attend at least one conference to present findings from this work.

For peer review only - http://bmjopen.bmj.com/site/about/guidelines.xhtml

References

1. Ghaferi AA, Osborne NH, Birkmeyer JD, Dimick JB. Hospital characteristics associated with failure to rescue from complications after pancreatectomy. J Am Coll Surg [Internet]. Elsevier Inc.; 2010;211(3):325–30. Available from: http://dx.doi.org/10.1016/j.jamcollsurg.2010.04.025

2. Ghaferi AA, Birkmeyer JD, Dimick JB. Hospital volume and failure to rescue with high-risk surgery. Med Care. 2011;49(12):1076–81.

3. NHS Improvement. The adult patient who is deteriorating: sharing learning from literature, incident reports and root cause analysis investigations. 2016;(July):23. Available from: https://improvement.nhs.uk/documents/176/Deterioration_in_adults_report_7july.pdf

4. Findlay G, Shotton H, Mason K. Time to Intervene? Natl Confid Enq into Patient Oucome Death [Internet]. 2012;1–125. Available from: http://www.ncepod.org.uk/2012report1/downloads/CAP_fullreport.pdf

5. Fox P. An acute problem. Nurs Elder. 2005;3(4):33.

6. Harper SJ. NCEPOD: Caring to the end? J Intensive Care Soc. 2010;11(2):88–9.

7. National Confidential Enquiry into Patient Outcome and Death. Emergency Admissions: A jouney in the right direction? 2007; Available from: http://www.ncepod.org.uk/2007ea.htm

8. NICE. Acutely ill adults in hospital: recognising Acutely ill adults in hospital: recognising and responding to deterior and responding to deterioration (CG50). 2007;(July). Available from: www.nice.org.uk/guidance/cg50

9. Johnston M, Arora S, King D, Stroman L, Darzi A. Escalation of care and failure to rescue: A multicenter, multiprofessional qualitative study. Surgery [Internet]. 2014 Jun [cited 2017 Aug 7];155(6):989–94. Available from:

For peer review only - http://bmjopen.bmj.com/site/about/guidelines.xhtml

http://linkinghub.elsevier.com/retrieve/pii/S0039606014000476

10. Chua WL, Mackey S, Ng EKC, Liaw SY. Front line nurses' experiences with deteriorating ward patients: A qualitative study. Int Nurs Rev. 2013;60(4):501–9.

11. Mochizuki K, Shintani R, Mori K, Sato T, Sakaguchi O, Takeshige K, et al. Importance of respiratory rate for the prediction of clinical deterioration after emergency department discharge: a single-center, case-control study. Acute Med Surg [Internet]. 2016;1–7. Available from: http://doi.wiley.com/10.1002/ams2.252

12. Brady PW, Goldenhar LM. A qualitative study examining the influences on situation awareness and the identification, mitigation and escalation of recognised patient risk. BMJ Qual Saf [Internet]. 2014 [cited 2017 May 9];23:153–61. Available from: http://dx.doi.org/10.1136/bmjqs-2012-001747

13. Shearer B, Marshall S, Buist MD, Finnigan M, Kitto S, Hore T, et al. What stops hospital clinical staff from following protocols? An analysis of the incidence and factors behind the failure of bedside clinical staff to activate the rapid response system in a multi-campus Australian metropolitan healthcare service. BMJ Qual Saf [Internet]. 2012;21(7):569–75. Available from: http://qualitysafety.bmj.com/lookup/doi/10.1136/bmjqs-2011-000692

14. Donohue LA, Endacott R. Track, trigger and teamwork: Communication of deterioration in acute medical and surgical wards Background. Intensive Crit Care Nurs [Internet]. 2010 [cited 2017 May 9];26. Available from: http://ac.els-cdn.com/S0964339709001074/1-s2.0-S0964339709001074-main.pdf?_tid=42ebcfc6-34aa-11e7-a31b-00000aacb362&acdnat=1494329333_5d17d9cc08ce503e7b37debb709eaa99

15. Human Factors in Healthcare Patient Stories. 2013;

16. Greenhalgh T. HOW TO READ The basics of evidence based medicine [Internet]. Vol. 315, British Medical Journal. 2001. 596-9 p. Available from:

For peer review only

http://www.pubmedcentral.nih.gov/articlerender.fcgi?artid=2127419&tool=pmcentrez&rendertype=abstract

17. Tacconelli E. Systematic reviews: CRD's guidance for undertaking reviews in health care [Internet]. Vol. 10, The Lancet Infectious Diseases. 2010. 226 p. Available from: http://linkinghub.elsevier.com/retrieve/pii/S1473309910700657

18. Lewin S, Booth A, Glenton C, Munthe-Kaas H, Rashidian A, Wainwright M, et al. Applying GRADE-CERQual to qualitative evidence synthesis findings: introduction to the series. Implement Sci [Internet]. 2018 [cited 2018 Dec 17];13(1). Available from: http://creativecommons.org/publicdomain/zero/1.0/

19. Barnett-Page E, Thomas J. Methods for the synthesis of qualitative research: A critical review. BMC Med Res Methodol. 2008;9(1).

20. Dixon-Woods M. Using framework-based synthesis for conducting reviews of qualitative studies. BMC Med. 2011;9:9–10.

21. Enhancing transparency in reporting the synthesis of qualitative research: ENTREQ | The EQUATOR Network [Internet]. [cited 2018 Dec 14]. Available from: http://www.equator-network.org/reporting-guidelines/entreq/

For peer review only

For peer review only - http://bmjopen.bmj.com/site/about/guidelines.xhtml

For peer review only

For peer review only - http://bmjopen.bmj.com/site/about/guidelines.xhtml

Contributions

PW is the guarantor. JE was responsible for the overall design of the QES. JE and VW drafted the manuscript. TP and JE developed the search strategy. PW and JD provided QES and qualitative expertise. All authors read, provided feedback and approved the final manuscript.

Declarations

Acknowledgement

We would like to thank patient representative, TD for his contribution to this work.

Funding

This QES protocol is funded by NIHR Biomedical Research Centre, based at Oxford University Hospitals Trust, Oxford and the Department of Health and Wellcome Trust through the Health Innovation Challenge Fund.

This publication presents independent research commissioned by the Health Innovation Challenge Fund (HICF-R9-524; WT-103703/Z/14/Z), a parallel funding partnership between the Department of Health and Wellcome Trust. The views expressed in this publication are those of the author(s) and not necessarily those of the Department of Health or Wellcome Trust. This work was also supported by the NIHR Biomedical Research Centre, Oxford.

Availability of data and materials

Not applicable

Consent for publication

Not applicable

Ethics approval and consent to participate

Not applicable

Competing interests

The authors declare that they have no competing interests.

For peer review only - http://bmjopen.bmj.com/site/about/guidelines.xhtml

Supplementary File 1 (Draft Search Strategy for MEDLINE)

1. HOSPITALIZATION/

2. TERTIARY CARE CENTERS/

3. (ward or wards).ab,ti.

4. (inhospital or inpatient* or "in hospital").ab,ti.

5. (hospitalised or hospitalized).ab,ti.

6. "general hospital".ab,ti.

7. "nurs* staff* ".ab,ti.

8. "in patient".ab,ti.

9. bedside.ab,ti.

10. outreach.ab,ti.

11. 1 or 2 or 3 or 4 or 5 or 6 or 7 or 8 or 9 or 10

12. FAILURE TO RESCUE,HEALTH CARE/

13. "fail* to rescue ".ab,ti.

14. VITAL SIGNS/

15. MONITORING,PHYSIOLOGIC/

16. "vital sign* ".ab,ti.

17. (track and trigger).ab,ti.

18."early warning".ab,ti.

19. "warning score* ".ab,ti.

20. "early sign* ".ab,ti.

21. "warning system* ".ab,ti.

22. (deteriorat* or escalat*).ab,ti.

23. triggering.ab,ti.

24. HOSPITAL RAPID RESPONSE TEAM/

25. "rapid response".ab,ti.

26. "critical care outreach".ab,ti.

27. 12 or 13 or 14 or 15 or 16 or 17 or 18 or 19 or 20 or 21 or 22 or 23 or 24 or 25 or 26

28. MEDICAL ERROR/

29. DELAYED DIAGNOSIS/

30. COMMUNICATION/

31. PATIENT CARE TEAMS/

For peer review only

32. PATIENT SAFETY/

33. ORGANIZATIONAL CULTURE/

34. LEADERSHIP/

35. "human factor* ".af.

36. "human error* ".af.

37. "clinical error* ".af.

38. "medical error* ".af.

39. "protocol adherence".af.

40. "protocol compliance".af.

41. "teamwork*".af.

42. communication.af.

43. ("socio cultural" or sociocultural).af.

44. "situation awareness".af.

45. "organisational culture".af.

46. "organizational culture".af.

47. "safety culture".af.

48. "patient safety".af.

49. leadership.af.

50. "root cause analysis".af.

51. 28 or 29 or 30 or 31 or 32 or 33 or 34 or 35 or 36 or 37 or 38 or 39 or 40 or 41 or 42 or 43 or 44 or 45 or 46 or 47 or 48 or 49 or 50

52. 11 and 27 and 51

For peer review only

PRISMA-P (Preferred Reporting Items for Systematic review and Meta-Analysis Protocols) 2015 checklist: recommended items to address in a systematic review protocol*

Section and topic	Item No	Checklist item	Page No
**ADMINISTRATIVE INFORMATION**			
Title:			
Identification	1a	Identify the report as a protocol of a systematic review	Pg 1 Line 3
Update	1b	If the protocol is for an update of a previous systematic review, identify as such	n/a
Registration	2	If registered, provide the name of the registry (such as PROSPERO) and registration number	Pg 2 Line 29<br>Pg 3 Lines 25-26
Authors:			
Contact	3a	Provide name, institutional affiliation, e-mail address of all protocol authors; provide physical mailing address of corresponding author	Pg 1 Lines 6-10
Contributions	3b	Describe contributions of protocol authors and identify the guarantor of the review	Pg 11 Lines 1-5
Amendments	4	If the protocol represents an amendment of a previously completed or published protocol, identify as such and list changes; otherwise, state plan for documenting important protocol amendments	n/a
Support:			
Sources	5a	Indicate sources of financial or other support for the review	Pg 1 Lines 13-16<br>Pg 11 Lines 7-15
Sponsor	5b	Provide name for the review funder and/or sponsor	Pg 1 Lines 13-16<br>Pg 11 Lines 7-15
Role of sponsor or funder	5c	Describe roles of funder(s), sponsor(s), and/or institution(s), if any, in developing the protocol	n/a
**INTRODUCTION**			
Rationale	6	Describe the rationale for the review in the context of what is already known	Pg 2 Lines 2-12<br>Pg 3 Lines 1-14
Objectives	7	Provide an explicit statement of the question(s) the review will address with reference to participants, interventions, comparators, and outcomes (PICO)	Pg 3 Lines 15-20
**METHODS**			
Eligibility criteria	8	Specify the study characteristics (such as PICO, study design, setting, time frame) and report characteristics (such as years considered, language, publication status) to be used as criteria for eligibility for the review	Pg 3 Lines 34-36<br>Pg 4 Lines 1-18

For peer review only

Information sources	9	Describe all intended information sources (such as electronic databases, contact with study authors, trial registers or other grey literature sources) with planned dates of coverage	Pg 3 Lines 27-32
Search strategy	10	Present draft of search strategy to be used for at least one electronic database, including planned limits, such that it could be repeated	Supplementary File 1
Study records:			
Data management	11a	Describe the mechanism(s) that will be used to manage records and data throughout the review	Pg 4 Lines 36-38<br>Pg 5 Lines 4-8
Selection process	11b	State the process that will be used for selecting studies (such as two independent reviewers) through each phase of the review (that is, screening, eligibility and inclusion in meta-analysis)	Pg 4 Lines 38-44
Data collection process	11c	Describe planned method of extracting data from reports (such as piloting forms, done independently, in duplicate), any processes for obtaining and confirming data from investigators	Pg 5 Lines 4-12
Data items	12	List and define all variables for which data will be sought (such as PICO items, funding sources), any pre-planned data assumptions and simplifications	Pg 4 Lines 7-10(inc table)
Outcomes and prioritization	13	List and define all outcomes for which data will be sought, including prioritization of main and additional outcomes, with rationale	Pg 4 Lines 9-10
Risk of bias in individual studies	14	Describe anticipated methods for assessing risk of bias of individual studies, including whether this will be done at the outcome or study level, or both; state how this information will be used in data synthesis	Pg 5 Lines 15-28
Data synthesis	15a	Describe criteria under which study data will be quantitatively synthesised	n/a
	15b	If data are appropriate for quantitative synthesis, describe planned summary measures, methods of handling data and methods of combining data from studies, including any planned exploration of consistency (such as I^2, Kendall's τ)	n/a
	15c	Describe any proposed additional analyses (such as sensitivity or subgroup analyses, meta-regression)	n/a
	15d	If quantitative synthesis is not appropriate, describe the type of summary planned	n/a
Meta-bias(es)	16	Specify any planned assessment of meta-bias(es) (such as publication bias across studies, selective reporting within studies)	Pg 5 Lines 15-28
Confidence in cumulative evidence	17	Describe how the strength of the body of evidence will be assessed (such as GRADE)	Pg 5 Lines 22-28

***It is strongly recommended that this checklist be read in conjunction with the PRISMA-P Explanation and Elaboration (cite when available) for important clarification on the items. Amendments to a review protocol should be tracked and dated. The copyright for PRISMA-P (including checklist) is held by the PRISMA-P Group and is distributed under a Creative Commons Attribution Licence 4.0.**

From: Shamseer L, Moher D, Clarke M, Ghersi D, Liberati A, Petticrew M, Shekelle P, Stewart L, PRISMA-P Group. Preferred reporting items for systematic review and meta-analysis protocols (PRISMA-P) 2015: elaboration and explanation. BMJ. 2015 Jan 2;349(jan02 1):g7647.

For peer review only
